# 5-Bromo-2-methyl-3-(4-methyl­phenyl­sulfon­yl)-1-benzofuran

**DOI:** 10.1107/S1600536812026918

**Published:** 2012-06-20

**Authors:** Hong Dae Choi, Pil Ja Seo, Uk Lee

**Affiliations:** a Department of Chemistry, Dongeui University, San 24 Kaya-dong, Busanjin-gu, Busan 614-714, Republic of Korea; b Department of Chemistry, Pukyong National University, 599-1 Daeyeon 3-dong, Nam-gu, Busan 608-737, Republic of Korea

## Abstract

In the title compound, C_16_H_13_BrO_3_S, the 4-methyl­phenyl group makes a dihedral angle of 70.1 (1)° with the mean plane [average deviation = 0.012 (2) Å] of the benzofuran unit. In the crystal, mol­ecules are linked by weak C—H⋯O hydrogen bonds. There are also π–π inter­actions between the furan and benzene rings of adjacent benzofuran systems [centroid–centroid distances = 3.621 (2) and 3.665 (2) Å], along with halogen–halogen inter­actions [Br⋯Br separation = 3.674 (1) Å].

## Related literature

For background information and the crystal structures of related compounds, see: Choi *et al.* (2008 ▶, 2010 ▶).
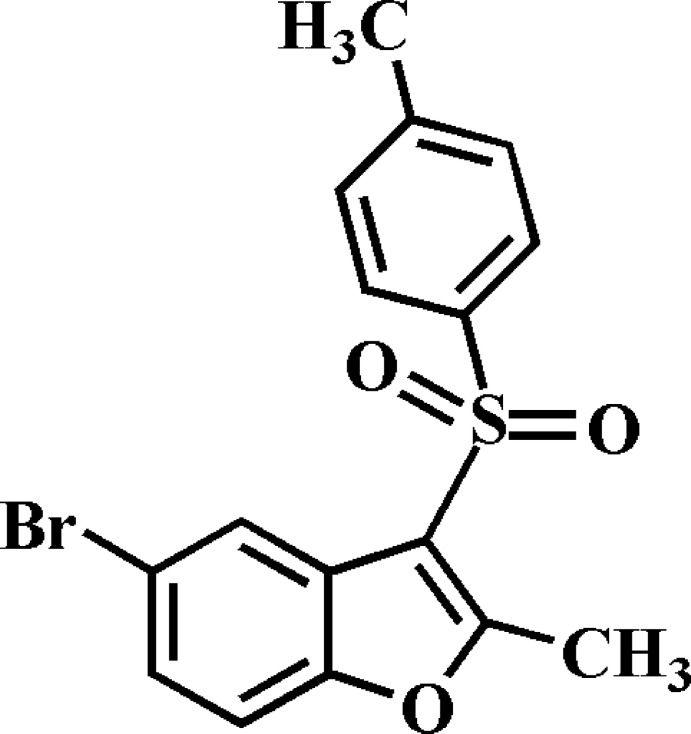


## Experimental

### 

#### Crystal data

C_16_H_13_BrO_3_S
*M* *_r_* = 365.23
Triclinic, 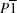

*a* = 7.2126 (2) Å
*b* = 10.3607 (3) Å
*c* = 11.1748 (3) Å
α = 112.987 (2)°
β = 90.340 (2)°
γ = 107.903 (2)°
*V* = 723.93 (3) Å^3^
*Z* = 2
Mo *K*α radiation
μ = 2.99 mm^−1^
*T* = 173 K
0.24 × 0.16 × 0.14 mm

#### Data collection

Bruker SMART APEXII CCD diffractometer
Absorption correction: multi-scan (*SADABS*; Bruker, 2009 ▶) *T*_min_ = 0.563, *T*_max_ = 0.746
13654 measured reflections
3618 independent reflections
2977 reflections with *I* > 2σ(*I*)
*R*_int_ = 0.037

#### Refinement

*R*[*F* ^2^ > 2σ(*F* ^2^)] = 0.034
*wR*(*F* ^2^) = 0.087
*S* = 1.03
3618 reflections
192 parameters
H-atom parameters constrained
Δρ_max_ = 0.61 e Å^−3^
Δρ_min_ = −0.38 e Å^−3^

### 

Data collection: *APEX2* (Bruker, 2009 ▶); cell refinement: *SAINT* (Bruker, 2009 ▶); data reduction: *SAINT*; program(s) used to solve structure: *SHELXS97* (Sheldrick, 2008 ▶); program(s) used to refine structure: *SHELXL97* (Sheldrick, 2008 ▶); molecular graphics: *ORTEP-3* (Farrugia, 1997 ▶) and *DIAMOND* (Brandenburg, 1998 ▶); software used to prepare material for publication: *SHELXL97*.

## Supplementary Material

Crystal structure: contains datablock(s) global, I. DOI: 10.1107/S1600536812026918/ld2064sup1.cif

Structure factors: contains datablock(s) I. DOI: 10.1107/S1600536812026918/ld2064Isup2.hkl

Supplementary material file. DOI: 10.1107/S1600536812026918/ld2064Isup3.cml

Additional supplementary materials:  crystallographic information; 3D view; checkCIF report

## Figures and Tables

**Table 1:** Hydrogen-bond geometry (Å, °)

*D*—H⋯*A*	*D*—H	H⋯*A*	*D*⋯*A*	*D*—H⋯*A*
C11—H11⋯O3^i^	0.95	2.52	3.236 (3)	132
C15—H15⋯O2^ii^	0.95	2.41	3.287 (3)	153

Symmetry codes: (i) 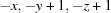; (ii) 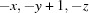.

**Fig. 1.**
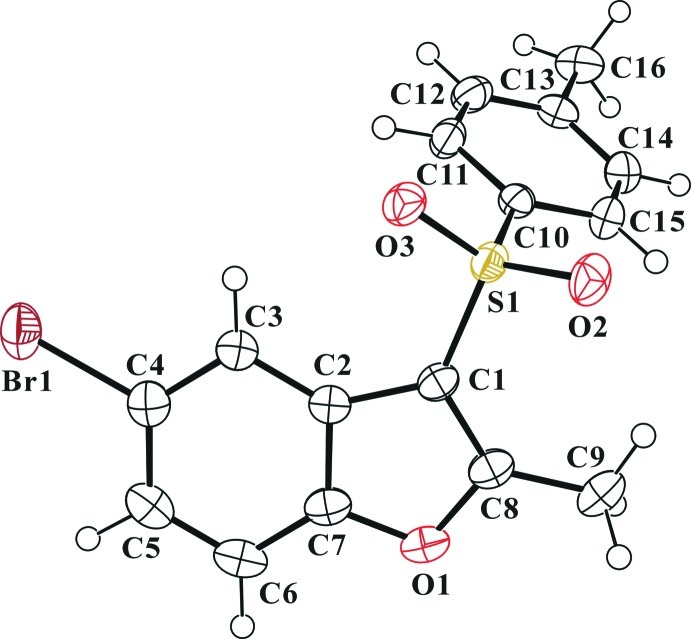
The molecular structure of the title compound with the atom numbering scheme. Displacement ellipsoids are drawn at the 50% probability level. H atoms are presented as small spheres of arbitrary radius.

**Fig. 2.**
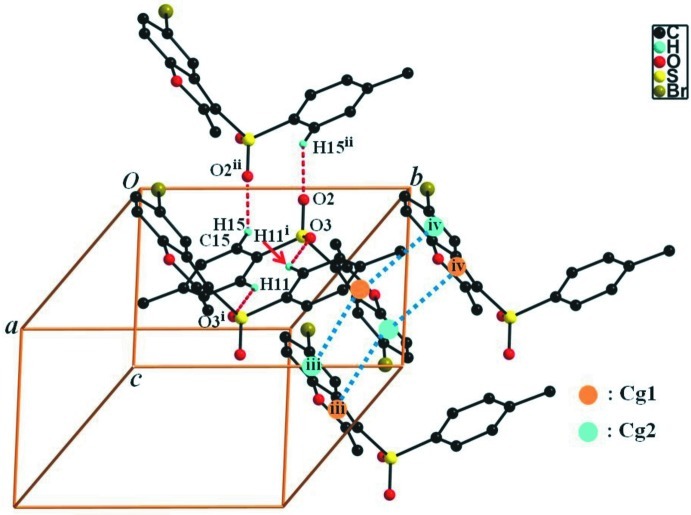
A view of the C—H···O and π–π interactions (dotted lines) in the crystal structure of the title compound. H atoms non-participating in the hydrogen-bonding were omitted for clarity. [Symmetry codes: (i) - x, - y + 1, - z + 1; (ii) - x, - y + 1, - z; (iii) - x + 1, - y + 2, - z + 1; (iv) - x, - y + 2, - z + 1.]

## supplementary crystallographic information

### Comment

As a part of our ongoing study of 5-bromo-2-methyl-1-benzofuran derivatives containing 3-phenylsulfonyl (Choi *et al.*, 2008) and 3-(4-fluorophenylsulfonyl) (Choi *et al.*, 2010) substituents, we report herein the crystal structure of the title compound.

In the title molecule (Fig. 1), the benzofuran unit is essentially planar, with a mean deviation of 0.012 (2) Å from the least-squares plane defined by the nine constituent atoms. The dihedral angle between the 4-methylphenyl ring and the mean plane of the benzofurn fragment is 70.09 (6) °. In the crystal structure (Fig. 2), molecules are connected by weak C—H···O hydrogen bonds (Table1). The crystal packing (Fig. 2) also exhibits slipped π–π interactions between the furan and benzene rings of adjacent benzofuran systems, with Cg1···Cg2^iii^ & Cg1···Cg2^iv^ distances of 3.621 (2) Å & 3.665 (2) Å and interplanar distances of 3.542 (2) Å & 3.408 (2) Å resulting in slippages of 0.752 (2) Å & 1.348 (2) Å (Cg1 and Cg2 are the centroids of the C1/C2/C7/O1/C8 furan ring and the C2-C7 benzene ring, respectively).The crystal packing also exhibits a Br···Br^i^ contact at 3.6736 (5) Å [(i) - *x*, - *y* + 2, - *z* + 2].

### Experimental

3-Chloroperoxybenzoic acid (77%, 448 mg, 2.0 mmol) was added in small portions to a stirred solution of 5-bromo-2-methyl-3-(4-methylphenylsulfanyl)-1-benzofuran (300 mg, 0.9 mmol) in dichloromethane (40 mL) at 273 K. After being stirred at room temperature for 10h, the mixture was washed with saturated sodium bicarbonate solution, and the organic layer was separated, dried over magnesium sulfate, filtered and concentrated at reduced pressure. The residue was purified by column chromatography (benzene) to afford the title compound as a colorless solid [yield 73%, m.p. 468–469 K; *R*_f_ = 0.65 (benzene)]. Single crystals suitable for X-ray diffraction were prepared by slow evaporation of a solution of the title compound in ethyl acetate at room temperature.

### Refinement

All H atoms were positioned geometrically and refined using a riding model, with C—H = 0.95 Å for aryl and 0.98 Å for methyl H atoms. Uiso(H) = 1.2*U*_eq_(C) for aryl and 1.5*U*_eq_(C) for methyl H atoms. The positions of methyl hydrogens were optimized rotationally.

### Crystal data

**Table:**

C_16_H_13_BrO_3_S	*Z* = 2
*M**_r_* = 365.23	*F*(000) = 368
Triclinic, *P*1	*D*_x_ = 1.676 Mg m^−^^3^
Hall symbol: -P 1	Mo *K*α radiation, λ = 0.71073 Å
*a* = 7.2126 (2) Å	Cell parameters from 5035 reflections
*b* = 10.3607 (3) Å	θ = 3.0–28.0°
*c* = 11.1748 (3) Å	µ = 2.99 mm^−^^1^
α = 112.987 (2)°	*T* = 173 K
β = 90.340 (2)°	Block, colourless
γ = 107.903 (2)°	0.24 × 0.16 × 0.14 mm
*V* = 723.93 (3) Å^3^	

### Data collection

**Table:**

Bruker SMART APEXII CCD diffractometer	3618 independent reflections
Radiation source: rotating anode	2977 reflections with *I* > 2σ(*I*)
Graphite multilayer monochromator	*R*_int_ = 0.037
Detector resolution: 10.0 pixels mm^-1^	θ_max_ = 28.4°, θ_min_ = 2.0°
φ and ω scans	*h* = −9→9
Absorption correction: multi-scan (*SADABS*; Bruker, 2009)	*k* = −13→13
*T*_min_ = 0.563, *T*_max_ = 0.746	*l* = −14→14
13654 measured reflections	

### Refinement

**Table:**

Refinement on *F*^2^	Primary atom site location: structure-invariant direct methods
Least-squares matrix: full	Secondary atom site location: difference Fourier map
*R*[*F*^2^ > 2σ(*F*^2^)] = 0.034	Hydrogen site location: difference Fourier map
*wR*(*F*^2^) = 0.087	H-atom parameters constrained
*S* = 1.03	*w* = 1/[σ^2^(*F*_o_^2^) + (0.041*P*)^2^ + 0.3896*P*] where *P* = (*F*_o_^2^ + 2*F*_c_^2^)/3
3618 reflections	(Δ/σ)_max_ = 0.001
192 parameters	Δρ_max_ = 0.61 e Å^−^^3^
0 restraints	Δρ_min_ = −0.38 e Å^−^^3^

### Special details

**Table:**

Geometry. All esds (except the esd in the dihedral angle between two l.s. planes) are estimated using the full covariance matrix. The cell esds are taken into account individually in the estimation of esds in distances, angles and torsion angles; correlations between esds in cell parameters are only used when they are defined by crystal symmetry. An approximate (isotropic) treatment of cell esds is used for estimating esds involving l.s. planes.
Refinement. Refinement of F^2^ against ALL reflections. The weighted R-factor wR and goodness of fit S are based on F^2^, conventional R-factors R are based on F, with F set to zero for negative F^2^. The threshold expression of F^2^ > 2sigma(F^2^) is used only for calculating R-factors(gt) etc. and is not relevant to the choice of reflections for refinement. R-factors based on F^2^ are statistically about twice as large as those based on F, and R- factors based on ALL data will be even larger.

### Fractional atomic coordinates and isotropic or equivalent isotropic displacement parameters (Å^2^)

**Table:**

	*x*	*y*	*z*	*U*_iso_*/*U*_eq_	
Br1	0.15488 (4)	1.00069 (3)	0.87021 (2)	0.04424 (11)	
S1	−0.00690 (8)	0.60478 (6)	0.27695 (5)	0.02645 (13)	
O1	0.3145 (2)	1.01741 (18)	0.35296 (17)	0.0327 (4)	
O2	−0.1043 (2)	0.5621 (2)	0.14793 (15)	0.0359 (4)	
O3	−0.1223 (2)	0.58519 (18)	0.37716 (15)	0.0315 (4)	
C1	0.1341 (3)	0.7923 (2)	0.3393 (2)	0.0261 (4)	
C2	0.1767 (3)	0.8934 (2)	0.4763 (2)	0.0253 (4)	
C3	0.1348 (3)	0.8827 (3)	0.5943 (2)	0.0277 (5)	
H3	0.0613	0.7907	0.5969	0.033*	
C4	0.2051 (3)	1.0124 (3)	0.7073 (2)	0.0321 (5)	
C5	0.3109 (4)	1.1496 (3)	0.7075 (3)	0.0360 (5)	
H5	0.3537	1.2360	0.7879	0.043*	
C6	0.3537 (3)	1.1604 (3)	0.5906 (3)	0.0353 (5)	
H6	0.4257	1.2528	0.5881	0.042*	
C7	0.2871 (3)	1.0310 (3)	0.4783 (2)	0.0290 (5)	
C8	0.2210 (3)	0.8717 (3)	0.2700 (2)	0.0295 (5)	
C9	0.2361 (4)	0.8320 (3)	0.1296 (2)	0.0387 (6)	
H9A	0.3738	0.8462	0.1160	0.058*	
H9B	0.1888	0.8958	0.1008	0.058*	
H9C	0.1557	0.7276	0.0785	0.058*	
C10	0.1604 (3)	0.5089 (2)	0.2602 (2)	0.0261 (4)	
C11	0.2062 (3)	0.4715 (3)	0.3602 (2)	0.0300 (5)	
H11	0.1513	0.5010	0.4395	0.036*	
C12	0.3325 (4)	0.3911 (3)	0.3435 (2)	0.0334 (5)	
H12	0.3611	0.3630	0.4110	0.040*	
C13	0.4185 (3)	0.3503 (3)	0.2297 (2)	0.0317 (5)	
C14	0.3751 (4)	0.3926 (3)	0.1327 (2)	0.0353 (5)	
H14	0.4359	0.3679	0.0556	0.042*	
C15	0.2461 (4)	0.4695 (3)	0.1453 (2)	0.0334 (5)	
H15	0.2156	0.4956	0.0768	0.040*	
C16	0.5507 (4)	0.2586 (3)	0.2102 (3)	0.0420 (6)	
H16A	0.4759	0.1540	0.1549	0.063*	
H16B	0.6016	0.2675	0.2956	0.063*	
H16C	0.6609	0.2943	0.1673	0.063*	

### Atomic displacement parameters (Å^2^)

**Table:**

	*U*^11^	*U*^22^	*U*^33^	*U*^12^	*U*^13^	*U*^23^
Br1	0.05295 (18)	0.05149 (18)	0.02791 (13)	0.02311 (14)	0.00691 (11)	0.01176 (11)
S1	0.0248 (3)	0.0301 (3)	0.0224 (2)	0.0043 (2)	0.00156 (19)	0.0125 (2)
O1	0.0310 (8)	0.0322 (9)	0.0430 (9)	0.0105 (7)	0.0085 (7)	0.0237 (7)
O2	0.0321 (8)	0.0457 (10)	0.0261 (8)	0.0068 (8)	−0.0045 (6)	0.0159 (7)
O3	0.0286 (8)	0.0337 (9)	0.0298 (8)	0.0040 (7)	0.0058 (6)	0.0155 (7)
C1	0.0242 (10)	0.0293 (11)	0.0292 (10)	0.0098 (9)	0.0043 (8)	0.0160 (9)
C2	0.0201 (9)	0.0261 (11)	0.0306 (10)	0.0082 (8)	0.0030 (8)	0.0124 (9)
C3	0.0249 (10)	0.0278 (12)	0.0300 (10)	0.0082 (9)	0.0023 (8)	0.0119 (9)
C4	0.0295 (11)	0.0361 (13)	0.0320 (11)	0.0142 (10)	0.0039 (9)	0.0130 (10)
C5	0.0319 (12)	0.0293 (12)	0.0410 (13)	0.0117 (10)	−0.0017 (10)	0.0075 (10)
C6	0.0279 (11)	0.0257 (12)	0.0518 (14)	0.0080 (10)	0.0014 (10)	0.0163 (11)
C7	0.0234 (10)	0.0310 (12)	0.0392 (12)	0.0113 (9)	0.0052 (9)	0.0194 (10)
C8	0.0248 (10)	0.0342 (12)	0.0358 (11)	0.0109 (9)	0.0050 (9)	0.0202 (10)
C9	0.0404 (13)	0.0507 (16)	0.0388 (13)	0.0173 (12)	0.0127 (11)	0.0308 (12)
C10	0.0279 (10)	0.0217 (10)	0.0239 (9)	0.0023 (9)	−0.0010 (8)	0.0093 (8)
C11	0.0319 (11)	0.0341 (12)	0.0241 (10)	0.0079 (10)	0.0050 (8)	0.0145 (9)
C12	0.0358 (12)	0.0340 (13)	0.0334 (11)	0.0078 (10)	0.0017 (10)	0.0200 (10)
C13	0.0290 (11)	0.0224 (11)	0.0372 (12)	0.0027 (9)	0.0017 (9)	0.0103 (9)
C14	0.0428 (13)	0.0333 (13)	0.0272 (11)	0.0122 (11)	0.0090 (10)	0.0102 (10)
C15	0.0414 (13)	0.0356 (13)	0.0228 (10)	0.0109 (11)	0.0037 (9)	0.0130 (9)
C16	0.0402 (14)	0.0336 (14)	0.0546 (16)	0.0122 (11)	0.0097 (12)	0.0209 (12)

### Geometric parameters (Å, º)

**Table:**

Br1—Br1^i^	3.6736 (5)	C8—C9	1.472 (3)
Br1—C4	1.901 (2)	C9—H9A	0.9800
S1—O2	1.4376 (16)	C9—H9B	0.9800
S1—O3	1.4395 (16)	C9—H9C	0.9800
S1—C1	1.736 (2)	C10—C11	1.385 (3)
S1—C10	1.753 (2)	C10—C15	1.397 (3)
O1—C8	1.366 (3)	C11—C12	1.381 (4)
O1—C7	1.374 (3)	C11—H11	0.9500
C1—C8	1.363 (3)	C12—C13	1.390 (3)
C1—C2	1.442 (3)	C12—H12	0.9500
C2—C3	1.392 (3)	C13—C14	1.384 (3)
C2—C7	1.395 (3)	C13—C16	1.503 (4)
C3—C4	1.380 (3)	C14—C15	1.375 (4)
C3—H3	0.9500	C14—H14	0.9500
C4—C5	1.391 (4)	C15—H15	0.9500
C5—C6	1.382 (4)	C16—H16A	0.9800
C5—H5	0.9500	C16—H16B	0.9800
C6—C7	1.373 (3)	C16—H16C	0.9800
C6—H6	0.9500		
			
C4—Br1—Br1^i^	152.33 (7)	O1—C8—C9	116.2 (2)
O2—S1—O3	119.55 (10)	C8—C9—H9A	109.5
O2—S1—C1	108.57 (11)	C8—C9—H9B	109.5
O3—S1—C1	106.20 (10)	H9A—C9—H9B	109.5
O2—S1—C10	107.83 (10)	C8—C9—H9C	109.5
O3—S1—C10	108.00 (10)	H9A—C9—H9C	109.5
C1—S1—C10	105.93 (10)	H9B—C9—H9C	109.5
C8—O1—C7	107.36 (17)	C11—C10—C15	120.3 (2)
C8—C1—C2	107.5 (2)	C11—C10—S1	120.07 (18)
C8—C1—S1	127.18 (18)	C15—C10—S1	119.65 (18)
C2—C1—S1	125.28 (16)	C12—C11—C10	119.4 (2)
C3—C2—C7	119.3 (2)	C12—C11—H11	120.3
C3—C2—C1	136.0 (2)	C10—C11—H11	120.3
C7—C2—C1	104.72 (19)	C11—C12—C13	121.2 (2)
C4—C3—C2	116.8 (2)	C11—C12—H12	119.4
C4—C3—H3	121.6	C13—C12—H12	119.4
C2—C3—H3	121.6	C14—C13—C12	118.4 (2)
C3—C4—C5	123.3 (2)	C14—C13—C16	120.6 (2)
C3—C4—Br1	117.80 (18)	C12—C13—C16	121.0 (2)
C5—C4—Br1	118.86 (18)	C15—C14—C13	121.6 (2)
C6—C5—C4	120.0 (2)	C15—C14—H14	119.2
C6—C5—H5	120.0	C13—C14—H14	119.2
C4—C5—H5	120.0	C14—C15—C10	119.1 (2)
C7—C6—C5	116.8 (2)	C14—C15—H15	120.5
C7—C6—H6	121.6	C10—C15—H15	120.5
C5—C6—H6	121.6	C13—C16—H16A	109.5
C6—C7—O1	126.0 (2)	C13—C16—H16B	109.5
C6—C7—C2	123.8 (2)	H16A—C16—H16B	109.5
O1—C7—C2	110.19 (19)	C13—C16—H16C	109.5
C1—C8—O1	110.2 (2)	H16A—C16—H16C	109.5
C1—C8—C9	133.6 (2)	H16B—C16—H16C	109.5
			
O2—S1—C1—C8	−29.7 (2)	C1—C2—C7—O1	−0.8 (2)
O3—S1—C1—C8	−159.5 (2)	C2—C1—C8—O1	−0.8 (3)
C10—S1—C1—C8	85.8 (2)	S1—C1—C8—O1	178.09 (16)
O2—S1—C1—C2	148.94 (19)	C2—C1—C8—C9	177.9 (3)
O3—S1—C1—C2	19.2 (2)	S1—C1—C8—C9	−3.2 (4)
C10—S1—C1—C2	−95.5 (2)	C7—O1—C8—C1	0.3 (2)
C8—C1—C2—C3	−178.9 (3)	C7—O1—C8—C9	−178.7 (2)
S1—C1—C2—C3	2.2 (4)	O2—S1—C10—C11	−150.10 (18)
C8—C1—C2—C7	1.0 (2)	O3—S1—C10—C11	−19.6 (2)
S1—C1—C2—C7	−177.92 (17)	C1—S1—C10—C11	93.82 (19)
C7—C2—C3—C4	0.8 (3)	O2—S1—C10—C15	29.5 (2)
C1—C2—C3—C4	−179.3 (2)	O3—S1—C10—C15	159.94 (17)
C2—C3—C4—C5	0.9 (4)	C1—S1—C10—C15	−86.62 (19)
C2—C3—C4—Br1	−178.74 (16)	C15—C10—C11—C12	−2.0 (3)
C3—C4—C5—C6	−1.3 (4)	S1—C10—C11—C12	177.55 (17)
Br1—C4—C5—C6	178.33 (18)	C10—C11—C12—C13	1.7 (3)
C4—C5—C6—C7	0.0 (4)	C11—C12—C13—C14	0.2 (3)
C5—C6—C7—O1	−179.7 (2)	C11—C12—C13—C16	−178.0 (2)
C5—C6—C7—C2	1.7 (4)	C12—C13—C14—C15	−1.8 (4)
C8—O1—C7—C6	−178.4 (2)	C16—C13—C14—C15	176.4 (2)
C8—O1—C7—C2	0.4 (2)	C13—C14—C15—C10	1.5 (4)
C3—C2—C7—C6	−2.1 (3)	C11—C10—C15—C14	0.4 (3)
C1—C2—C7—C6	177.9 (2)	S1—C10—C15—C14	−179.15 (18)
C3—C2—C7—O1	179.09 (19)		

Symmetry code: (i) −*x*, −*y*+2, −*z*+2.

### Hydrogen-bond geometry (Å, º)

**Table:**

*D*—H···*A*	*D*—H	H···*A*	*D*···*A*	*D*—H···*A*
C11—H11···O3^ii^	0.95	2.52	3.236 (3)	132
C15—H15···O2^iii^	0.95	2.41	3.287 (3)	153

Symmetry codes: (ii) −*x*, −*y*+1, −*z*+1; (iii) −*x*, −*y*+1, −*z*.
